# Psychological distress among people with probable COVID-19 infection: analysis of the UK Household Longitudinal Study

**DOI:** 10.1192/bjo.2021.63

**Published:** 2021-05-18

**Authors:** Claire L. Niedzwiedz, Michaela Benzeval, Kirsten Hainey, Alastair H. Leyland, Srinivasa Vittal Katikireddi

**Affiliations:** Institute of Health & Wellbeing, University of Glasgow, UK; Institute for Social and Economic Research, University of Essex, UK; MRC/CSO Social & Public Health Sciences Unit, University of Glasgow, UK; MRC/CSO Social & Public Health Sciences Unit, University of Glasgow, UK; MRC/CSO Social & Public Health Sciences Unit, University of Glasgow, UK;

**Keywords:** Epidemiology, COVID-19, depressive disorders, infectious disease, mental health

## Abstract

Studies exploring the longer-term effects of experiencing coronavirus disease-2019 (COVID-19) on mental health are lacking. We explored the relationship between reporting probable COVID-19 symptoms in April 2020 and psychological distress (measured using the General Health Questionnaire) 1, 2, 3, 5 and 7 months later. Data were taken from the UK Household Longitudinal Study, a nationally representative household panel survey of UK adults. Elevated levels of psychological distress were found up to 7 months after probable COVID-19, compared with participants with no likely infection. Associations were stronger among younger age groups and men. Further research into the psychological sequalae of COVID-19 is urgently needed.

## Background

Considerable concerns exist about the longer-term effects of experiencing coronavirus disease-2019 (COVID-19).^1^ However, population-based data remain rare, with the majority of COVID-19 research focused on severe adverse physical consequences of acute disease. There is growing evidence that a substantial number of people experience persisting symptoms such as fatigue and chest pain months after infection.^2^ Although the mental health consequences of societal changes during the pandemic (including lockdown) have been extensively studied,^3,4^ research on the impact of COVID-19 infection on mental health is limited.

Research from previous coronavirus outbreaks demonstrates potential for psychiatric consequences of infection.^5^ An initial study using administrative data from the USA demonstrated that COVID-19 infection was associated with an increased incidence of psychiatric diagnoses (particularly anxiety and insomnia) in the following 14–90 days.^6^ Studies have also demonstrated that COVID-19 survivors who received emergency department evaluation in Milan had high levels of depression and anxiety 1 month following discharge^7^ and COVID-19 patients in Chongqing, China had more symptoms of stress, anxiety and depression compared with healthy and psychiatric patient controls.^8^ However, most existing studies have been limited by small, non-representative samples and those based on electronic health records may not capture individuals with more minor acute COVID-19 symptoms or those with less severe psychological problems that do not present to health services. Changes in healthcare-seeking and treatment may lead to substantial potential bias in the context of a pandemic, where disruptions to health systems have been widespread. Data from surveys may therefore be better placed to capture the impact on mental health and need for intervention.

## Aims

To explore the relationship between COVID-19 infection and mental health in the UK context, we assessed associations between experiencing COVID-19 symptoms and changes in psychological distress in a representative longitudinal survey of adults.

## Method

The UK Household Longitudinal Study (also referred to as Understanding Society) is a nationally representative longitudinal household panel survey, based on a clustered stratified probability sample of UK households, described in detail previously.^9^ All adults (aged ≥16 years) in chosen households are invited to participate. Data collection for each wave usually takes place over 24 months, with participants re-interviewed every year by online, face-to-face or telephone survey.

We used pre-pandemic data from wave 9 (2017–19), which achieved a household response rate of over 80%.^10^ In response to the COVID-19 pandemic, additional waves of data were collected in 2020 via online survey during April (24 to 30 April), May (27 May to 2 June), June (25 June to 1 July), July (24 to 31 July), September (24 September to 1 October) and November (24 November to 1 December).^11^ The response rate for the first COVID wave was 48.6% of those who took part at wave 9.^12^

Psychological distress was measured at each wave (pre-pandemic in 2017–19 and during the pandemic at each wave as above) via the General Health Questionnaire 12-item instrument (GHQ-12).^13^ GHQ-12 assesses psychological distress and respondents reporting a score of 4 or more are likely experiencing symptoms to a clinically significant level.^3^ Self-reported symptoms of cough, fever and anosmia allowed identification of individuals with probable COVID-19 infection in April 2020 (see Supplementary Material available at https://doi.org/10.1192/bjo.2021.63).

Of 42 people reporting hospital admission for COVID-19 in April 2020, 34 were classified as probable COVID-19 according to our definition. We assessed associations between probable COVID-19 infection and psychological distress at 1, 2, 3, 5 and 7 months later using logistic regression, with models adjusted to account for pre-pandemic psychological distress (GHQ score) and other covariates (gender, age, ethnicity and long-standing illness or disability). We also conducted subgroup analyses by age group (under 45, 45–64, ≥65 years) and gender (men, women) to investigate potential effect modification by demographic characteristics associated with COVID-19.

Analyses used inverse probability weights to adjust for non-response and standard errors were adjusted for the complex survey design. Missing data were excluded from analyses, but if participants were missing pre-pandemic wave 9 data responses from wave 10 were used if available. Statistical analyses were performed in Stata/MP 15.1.

The University of Essex Ethics Committee approved all data collection for the Understanding Society main survey and COVID waves, which were performed in accordance with the Declaration of Helsinki. All survey participants provided fully informed consent. No additional ethical approval was necessary for this secondary data analysis.

## Results

In total, 8.9% (*n* = 1112) of 12 492 participants experienced probable COVID-19 symptoms in April 2020. Psychological distress was more prevalent in May (27.4%, 95% CI 25.9–28.9), reduced to 20.8% (95% CI 19.4–22.3) in July, before increasing again to 26.5% (95% CI 24.8–28.2) in November, corresponding with the UK winter lockdown. The characteristics of participants are described in Supplementary Table 1.

In comparison with participants without probable COVID-19 infection, psychological distress was more common at 1 (odds ratio (OR) = 1.39, 95% CI: 1.10–1.76), 2 (OR = 1.38, 95% CI 1.05–1.81), 3 (OR = 1.31, 95% CI 0.99–1.72), 5 (OR = 1.42, 95% CI 1.05–1.92) and 7 (OR = 1.47, 95% CI 1.04–2.07) months after reporting COVID-19 symptoms in adjusted analyses (Table 1 and Supplementary Table 2–6).

**Table 1:** Associations between probable coronavirus disease-2019 (COVID-19) in April 2020 and psychological distress (General Health Questionnaire (GHQ-12) ‘case’) up to 7 months later stratified by age group and gender^a^

	1 month later (May 2020)	2 months later (June 2020)	3 months later (July 2020)	5 months later (September 2020)	7 months later (November 2020)
Overall					
No likely infection (reference)	1	1	1	1	1
Probable infection,^b^ OR (95% CI)	1.39** (1.10–1.76)	1.38* (1.05–1.81)	1.31 (0.99–1.72)	1.42* (1.05–1.92)	1.47* (1.04–2.07)
*n*	12 492	11 949	11 563	11 009	10 379
By gender					
Men, OR (95% CI)	1.52* (1.05–2.20)	1.57* (1.04–2.37)	1.43 (0.91–2.24)	1.36 (0.88–2.12)	1.77** (1.20–2.61)
*n*	5138	4908	4759	4556	4283
Women, OR (95% CI)	1.30 (0.98–1.75)	1.31 (0.94–1.81)	1.25 (0.86–1.82)	1.46 (0.98–2.19)	1.30 (0.82–2.07)
*n*	7279	6969	6730	6379	6022
By age group					
Under 45 years, OR (95% CI)	1.51* (1.07–2.13)	1.43 (0.91–2.26)	1.20 (0.80–1.78)	1.39 (0.89–2.18)	1.74* (1.02–2.95)
*n*	3426	3169	2996	2735	2481
45–64 years, OR (95% CI)	1.24 (0.81–1.90)	1.39 (0.87–2.22)	1.38 (0.85–2.25)	1.53 (0.94–2.49)	1.27 (0.67–2.38)
*n*	5279	5045	4924	4724	4485
≥65 years, OR (95% CI)	1.16 (0.52–2.58)	1.14 (0.63–2.04)	1.33 (0.68–2.60)	1.23 (0.52–2.92)	1.14 (0.59–2.20)
*n*	3547	3511	3432	3329	3203

a. Results for each time point include people who participated in wave 9, April 2020 and the respective follow-up wave from May to November 2020.

b. Adjusted for age, gender, ethnicity, limiting long-standing illness, GHQ-12 at wave 9 (2017–19).

*P < 0.05, **P < 0.01, ***P < 0.001.

Subgroup analyses demonstrated stronger associations in men (OR = 1.52 in May, 95% CI 1.05–2.20) and younger adults (OR = 1.51 in May, 95% CI 1.07–2.13), with some differences in the strength of associations at different time points.

## Discussion

### Main findings

Our study suggests that COVID-19 infection could lead to an increase in clinically significant psychological distress that persists months after infection and is additional to the mental health impact of societal changes during the pandemic. Younger people and men with probable COVID-19 infection were more likely to report clinical levels of psychological distress compared with older age groups and women. There were some differences in the strength of associations over time that require further investigation to understand whether factors, such as lockdown restrictions, modify the association between COVID-19 symptoms and mental health.

### Strengths and limitations

Our findings add to the growing evidence that COVID-19 infection may have a direct impact on mental health^8,14^ that persists months after initial symptoms.^6,7^ Study strengths include the longitudinal data over multiple points of follow-up during the pandemic, the representative UK sample and accounting for pre-pandemic mental health to limit reverse causation. Important limitations include the ascertainment of COVID-19 infection, based on self-report only (not confirmed by a laboratory test) and the classification of probable COVID-19 infection at only one time point. However, misclassification may be more likely to result in underestimation of any underlying association. Further research based on confirmed infection is required, as is research that takes into account the fluctuating nature of symptoms (i.e. ‘long-COVID’) and research that compares the impact of COVID with that of other illnesses, such as pneumonia.

The self-reported nature of the exposure and outcome measures also means we cannot rule out the possibility of reverse causation in which poor mental health influences the reporting of COVID-19 symptoms. Triangulation of survey and administrative data (such as primary care and psychotropic prescribing records) would be helpful to disentangle the discrepancy between likely clinical need and service use and help to overcome the limitations of self-reported data.

### Implications

The potential adverse impact of COVID-19 infection on mental health reinforces the benefits of minimising COVID-19 infection among the general population, not only in those at greatest risk of mortality. When considered alongside the mental health impact generated by mitigation measures, there is potential for a high demand for mental health services resulting from the pandemic. Further research to examine the longer-term psychological sequelae of COVID-19 infection is urgently required.

## Data Availability

Understanding Society deidentified survey participant data are available through the UK Data Service (http://doi.org/10.5255/UKDA-SN-6614-14; http://doi.org/10.5255/UKDA-SN-8644-7). Researchers who would like to use Understanding Society need to register with the UK Data Service (https://ukdataservice.ac.uk/) before being allowed to download data-sets.

## Supplementary material

For supplementary material accompanying this paper visit http://dx.doi.org/10.1192/bjo.2021.63. click here to view supplementary material

## References

[1] Yelin D, Wirtheim E, Vetter P, Kalil AC, Bruchfeld J, Runold M, Long-term consequences of COVID-19: research needs. Lancet Infect Dis 2020; 20: 1115–7. 32888409 10.1016/S1473-3099(20)30701-5 PMC7462626

[2] Kingstone T, Taylor AK, Donnell CA, Atherton H, Blane DN, Chew-Graham CA. Finding the ‘right’ GP: a qualitative study of the experiences of people with long-COVID. BJGP Open 2020; 4: bjgpopen20X101143. 10.3399/bjgpopen20X101143 PMC7880173 33051223

[3] Niedzwiedz CL, Green MJ, Benzeval M, Campbell D, Craig P, Demou E, Mental health and health behaviours before and during the initial phase of the COVID-19 lockdown: longitudinal analyses of the UK Household Longitudinal Study. J Epidemiol Community Health 2021; 75:224–31. 32978210 10.1136/jech-2020-215060 PMC7892383

[4] O'Connor RC, Wetherall K, Cleare S, McClelland H, Melson AJ, Niedzwiedz CL, Mental health and wellbeing during the COVID-19 pandemic: longitudinal analyses of adults in the UK COVID-19 Mental Health & Wellbeing study. Br J Psychiatry [Epub ahead of print] 2020. Available from: 10.1192/bjp.2020.212. PMC7684009 33081860

[5] Rogers JP, Chesney E, Oliver D, Pollak TA, McGuire P, Fusar-Poli P, Psychiatric and neuropsychiatric presentations associated with severe coronavirus infections: a systematic review and meta-analysis with comparison to the COVID-19 pandemic. Lancet Psychiatry 2020; 7: 611–27. 32437679 10.1016/S2215-0366(20)30203-0 PMC7234781

[6] Taquet M, Luciano S, Geddes JR, Harrison PJ. Bidirectional associations between COVID-19 and psychiatric disorder: retrospective cohort studies of 62 354 COVID-19 cases in the USA. Lancet Psychiatry 2021; 8:130–40. 33181098 10.1016/S2215-0366(20)30462-4 PMC7820108

[7] Mazza MG, De Lorenzo R, Conte C, Poletti S, Vai B, Bollettini I, Anxiety and depression in COVID-19 survivors: role of inflammatory and clinical predictors. Brain Behav Immun 2020; 89: 594–600. 32738287 10.1016/j.bbi.2020.07.037 PMC7390748

[8] Hao F, Tam W, Hu X, Tan W, Jiang L, Jiang X, A quantitative and qualitative study on the neuropsychiatric sequelae of acutely ill COVID-19 inpatients in isolation facilities. Transl Psychiatry 2020; 10: 355. 33077738 10.1038/s41398-020-01039-2 PMC7570419

[9] University of Essex, Institute for Social and Economic Research. *Understanding Society: Waves 1-10, 2009-2019 and Harmonised BHPS: Waves 1-18, 1991-2009, User Guide*. University of Essex, 2020.

[10] University of Essex, Institute for Social and Economic Research. *Understanding Society: Waves 1-10, 2009-2019 and Harmonised BHPS: Waves 1-18, 1991-2009. [data collection]*. 13th ed. UK Data Service. SN: 6614, 2020. Available from: 10.5255/UKDA-SN-6614-14

[11] University of Essex, Institute for Social and Economic Research. *Understanding Society: COVID-19 Study. [data collection]*. 7th ed. UK Data Service. SN: 8644, 2021. Available from: 10.5255/UKDA-SN-8644-7

[12] Benzeval M, Burton J, Crossley TF, Fisher P, Jäckle A, Low H, The Idiosyncratic Impact of an Aggregate Shock: The Distributional Consequences of COVID-19. SSRN 2020. Available from: 10.2139/ssrn.3615691

[13] Goldberg DP, Gater R, Sartorius N, Ustun T B, Piccinelli M, Gureje O, The validity of two versions of the GHQ in the WHO study of mental illness in general health care. Psychol Med 1997; 27(1): 191–7. 9122299 10.1017/s0033291796004242

[14] Wang C, Pan R, Wan X, Tan Y, Xu L, McIntyre RS, A longitudinal study on the mental health of general population during the COVID-19 epidemic in China. Brain Behav Immun 2020; 87: 40–8. 32298802 10.1016/j.bbi.2020.04.028 PMC7153528

